# Rumdul (*Sphaerocoryne affinis*) Antioxidant Activity and Its Potential for Parkinson's Disease Treatment

**DOI:** 10.1155/2022/8918966

**Published:** 2022-03-18

**Authors:** Ngo Binh Thao Nghi, Tran Thuc Uyen, Huynh Man Anh, Dao My Linh, Dang Thi Phuong Thao

**Affiliations:** ^1^Faculty of Biology and Biotechnology, University of Science, Ho Chi Minh City, Vietnam; ^2^Vietnam National University, Ho Chi Minh City, Vietnam

## Abstract

Parkinson's disease (PD) is an age-related neurodegenerative disorder characterized by progressive deterioration of motor function and loss of dopaminergic neurons in the substantia nigra. Although PD is more common in people over 60 years old, people with young-onset PD tend to increase recently. Up to now, there is no cure for PD; therapies mainly focus on reducing symptoms and improving patient quality of life. Thus, the requirement of exploring new medications is needed. There is a strong relationship between oxidative stress and PD. Therefore, antioxidant compounds have been considered as a novel therapy for PD. In this study, we indicated a new potential candidate for PD treatment, rumdul fruit (*Sphaerocoryne affinis*—a member of the *Annonaceae* family), due to evaluating its activities on the fly model of Parkinson. Our experimental results showed that rumdul fruit water extract (RFWE) has a strong antioxidant capacity with IC50 value in DPPH assay which was 85.62 ± 1.05 *μ*g/mL. The use of RFWE at concentrations of 3, 6, and 12 mg/mL could strongly ameliorate the locomotor disabilities and dopaminergic neuron degeneration. Although the RFWE at high concentrations like 12 mg/mL and 18 mg/mL could induce some side effects on fly development and viability, our data strongly demonstrated that RFWE effectively rescued PD phenotypes on the fly model. Although component in the plant extract, as well as the molecular mechanism helping to recover the phenotype, has not been elucidated yet, the research contributed strong scientific evidence for further research on applying rumdul as a novel natural source for PD treatment.

## 1. Introduction

Parkinson's disease (PD) is an age-related neuron-degenerative disorder of the nervous system that was first described by James Parkinson, in 1917. PD is the second most common neuron disease, which impacts 2% of the population over 65 years old [1]. According to the WHO, the number of PD patients will increase two times in 2030 due to the intensified life span. Despite that, nowadays, there is no cure for PD. Recent therapies focus on relieving the symptoms and improving the life quality of patients. Therefore, many scientists have been making effort to gain insight into PD pathology and figure out new treatments.

It has been well-known that the combination of genetic and environmental factors involves in PD contribution. Some essential genes including *α-synuclein*, *LRRK2*, *PARKIN*, *DJ-1*, *PINK-1*, *GBA*, and *UCH-L1* were demonstrated to be associated with PD [2] [3]. Besides that, exposure with nerve agents, such as MPTP, 6-OHDA, rotenone, or paraquat, also promotes the risk of PD [4]. Many studies pointed out the strong relationship between oxidative stress and neurodegenerative diseases, including PD. High level of reactive oxygen species (ROS) was recognized in PD patients' substantia nigra which was demolished [5]. Therefore, antioxidant compounds have been considered as a novel therapy for PD [6]. However, utilizing one kind of antioxidant is possible to cause several side effects on physiology. For example, using high level of curcumin can lead to diarrhea, headache, rash, and yellow stool [7]. High intakes of vitamin C can induce kidney or stomach problems. Therefore, screening many sources of medicines for combining them to improve PD therapy efficiency is indispensable.

Several PD models have been used for screening and evaluating potential antioxidants for PD treatment. Among them, the *dUCH*-knocked down *Drosophila melanogaster* model has an advantage with high number of individuals in the population and can mimic the main PD phenotypes, including the defect in locomotor abilities and the progressive of DA neuron [8]. Besides that, antioxidant compounds such as vitamin C, curcumin, and vegetal such as Portulaca oleracea, Crocus sativus, and Ginkgo biloba also have been reported to mitigate the PD-like phenotypes in fly models [9] [10] [11].

On the other hand, rumdul (*Sphaerocoryne affinis*) is a flowering plant species and belongs to the *Annonaceae* family (soursop family), which originates from South–East Asia. Rumdul's leaves are long and flat with a size of 2-4 centimeters. The plant can grow up to 8-12 meters tall and measure 20-30 centimeters in diameter. Flowers of rumdul have been used in the cosmetic industry for a long time because of its fragrant flavor. Rumdul fruit growing in clusters has a sweet taste and purplish-black color, when ripe and soft [12]. In Vietnam, rumdul fruit can be used to produce juice and nondistilled alcoholic beverages [13]. Previous studies proposed that rumdul fruits have a great antioxidant capacity due to the high level of ascorbic acid and phenolic compounds [13]. However, the therapeutic activity of rumdul has not been clarified. Taken together, in this study, we aimed to evaluate the potential of the rumdul extract for PD treatment by using the *dUCH*-knockdown *Drosophila melanogaster* model.

## 2. Material and Methods

### 2.1. Fly Strains and Maintenance

Fly stocks were cultured on standard medium containing 5% dry yeast, 5% sucrose, 3% powdered milk, 0.1% sodium benzoate, 0.5% acid propionic, and 0.85% agar at 25°C [10]. For the rumdul fruit water extract (RFWE) medium, RFWE was added into the standard medium at final concentrations of 0, 3, 6, 12, and 18 mg/mL. The mixtures were placed away from direct light to restrain the degradation of antioxidant compounds.

GAL4-driver (TH-GAL4) was obtained from the Bloomington *Drosophila* stock center (code #8848) and was used to control the specific expression at the dopaminergic neurons of the target sequences. RNAi lines, including GD#26468 from the Vienna *Drosophila* Resource Center and 9331 from Bloomington Stock center, carried UAS-*dUCH*-IR sequence, the dsRNA of *dUCH* gene, and UAS-*GFP*-IR sequence, dsRNA of the *GFP* gene, in the order. Control and knockdown *dUCH* flies were generated by crossing the TH-GAL4 driver with the flies carrying UAS-*GFP*-IR and UAS-*dUCH*-IR, respectively. All flies used in the present study were treated at 28°C to increase the efficiency of the GAL4-UAS system.

### 2.2. Plant Material

The rumdul (*Sphaerocoryne affinis*) fruit was harvested in Tay Ninh province, Vietnam, in February 2017 with voucher specimen PHH0004912.

### 2.3. Preparing the Rumdul Fruit Water Extract (RFWE)

The protocol was conducted following the research of Truong et al. [11] with some modifications. Rumdul fruit was deep-frozen at -30°C and dried completely by freeze-drying. Then, rumdul dried fruit was ground into powder and carefully mixed with water in a ratio 1 : 5 (weight : volume) at room temperature. The mixture was incubated for 30 minutes at 4°C and centrifuged for 20 minutes at 7000 × g, 4°C. After that, the supernatant solution was collected, frozen at -30°C, and then completely dried again by freeze-drying. The aqueous rumdul extract powder was stored at -30°C for long-term preservation and used for further experiments. To restrict the degeneration of rumdul extraction's ingredients, the RFWE was limited from exposure to light, heat, and air.

### 2.4. DPPH Free Radical Scavenging Assay

The assay was performed based on the interaction between DPPH (1,1-diphenyl-2-picrylhydrazyl) (#D9132, Sigma, Singapore) and antioxidant compounds, which resulted in the discoloration of DPPH. The experiment was conducted following the protocol of Sharma and Bhat [14] with some modifications. Six concentrations of RFWE including 25, 50, 100, 200, 400, and 800 *μ*g/mL were utilized to establish the standard curve of RFWE antioxidant capacity. Vitamin C, L-ascorbic acid (#A0278-25G, Sigma, Singapore) used as a standard antioxidant substance was added to the reaction at final concentrations of 5, 10, 15, 20, and 25 *μ*M. Each reaction consisted of methanol solvent, 50 *μ*M DPPH, and different concentrations of sample. The blank for each sample contained methanol and distilled water, and the negative control composed of methanol, DPPH, and distilled water. All reactions were manipulated in a light-resistant condition for 40 minutes at 30°C. Then, the amount of the rest DPPH was measured using a spectrometer at 517 nm absorbance. The DPPH radical scavenging activity at each concentration was calculated following the formula[15]

(1) $$\begin{array}{c} \text{DPPH radical scavenging acitivity}=100-\frac{\text{Abs sample}-\text{Abs blank sample}}{\text{Abs negative control}}*100\; \end{array}$$

Then, the data were analyzed by GraphPad Prism 7.00 (GraphPad Software, USA) and used to calculate the half-maximal inhibitory concentration (IC_50_) based on accordant algorithms.

### 2.5. Feeding Assay

Feeding assay was operated to evaluate the food consumed by the larvae during a period. RFWE was used at concentrations of 0, 3, 6, 12, and 18 mg/mL. The assay was performed following a protocol described by Truong et al. [11] with some modifications as follows. 2% Coomassie Brilliant Blue G-250 (#808274-10 g, Biomedicals, USA) and RFWE supplemented medium were added in a 1.5 mL microcentrifuge tube. Then, the early third-instar larvae maintained on the respective medium were collected and transferred into tubes to consume Coomassie-containing medium within 30 minutes. After that, they were transferred into new tubes at a density of 10 larvae per tube, ground in PBS-10% ethanol by the pestle, and centrifuged at 10000 × g in 10 minutes. The supernatant was collected and used to evaluate the food intake by measuring the Coomassie level at 595 nm. Raw data were collected by Microsoft Excel 2016 (Microsoft, USA). The value was statistically analyzed and graphed using GraphPad Prism 7.00 (GraphPad Software, USA).

### 2.6. The Development and Toxicity

70 embryos of each fly strain were collected and transferred into standard medium containing RFWE at final concentrations of 0, 3, 6, 12, and 18 mg/mL. The numbers of new formed pupae and enclosed flies were daily counted. Raw data was collected by Microsoft Excel 2016 (Microsoft, USA). The average index was calculated, statistically analyzed, and graphed by GraphPad Prism 7.00 (GraphPad Software, USA).

### 2.7. Life Span Assay

This assay was performed as described previously with some modifications [11]. After being fed with standard medium containing RFWE, newly enclosed flies were anesthetized with diethyl ether and 100 male flies were randomly selected. These flies were continuously treated with RFWE and maintained at 28°C. The medium was renewed every 2-3 days. The number of dead flies was recorded one time per day at a fixed time. Raw data were collected by Microsoft Excel 2016 (Microsoft, USA). The value was statistically analyzed and graphed using GraphPad Prism 7.00.

### 2.8. Crawling Assay

Crawling assay was directly evaluated the movement ability of larvae by their crawling speed. The assay was performed following a previous protocol described by Truong et al. [11]. Male third-instar larvae were randomly chosen after being cultured with RFWE. Three of them were carefully put on 2% agar petri dish each time, and their movement was immediately recorded by a camera within a minute. These videos were analyzed by plugin wrMTrck of ImageJ software to receive average speed data. Raw data were collected by Microsoft Excel 2016 (Microsoft, USA). The value was calculated, statistically analyzed, and graphed using GraphPad Prism 7.00 (GraphPad Software, USA).

### 2.9. Immunostaining, Imaging, and DA Neuron Quantification

Immunofluorescence imaging and DA neuron quantification were performed following a protocol described by Truong et al. [11]. Tyrosine hydroxylase (TH) is a specific enzyme for dopaminergic neurons, which catalyzes for the converting of L-tyrosine to L-DOPA-dopamine precursor reaction. Therefore, it has been used as a marker to detect these neurons. Brains of third-instar larvae or adult flies were dissected in cold phosphate-buffered saline (PBS) and then fixed in 200 *μ*L 4% paraformaldehyde (PFA) at 25°C for 22 minutes. After that, these tissues were washed by 1 mL 0.3% PBS-Triton X-100 and blocked by the 100 *μ*L solution consisting of 10% goat serum (10 *μ*L) and 0.15% PBS-Triton X-100 (90 *μ*L) at 25 for 30 minutes. Brains were incubated with rabbit anti-tyrosine hydroxylase (anti-TH; Millipore, AB152, Japan), which was diluted in blocking solution (1 : 250) at 4°C for 36 hours. After being washed again with 1 mL 0.3% PBS-Triton X-100, brain tissues were incubated with secondary antibodies conjugated with Alexa 488 (1 : 500; Invitrogen) at 25°C for 2 hours. They were placed onto the slide after being washed, enclosed by Vectashield Mounting Medium (Vector Laboratories, Japan), and covered by a coverslip. The samples were observed by fluorescence microscope Nikon ECLIPSE NI-U. Images were analyzed using the cell counter plugin of ImageJ software. Raw data were collected by Microsoft Excel 2016 (Microsoft, USA). The value was statistically analyzed and graphed using GraphPad Prism 7.00 (GraphPad Software, USA).

## 3. Results

### 3.1. Antioxidant Capacity of Rumdul Fruit Water Extract

**Figure 1:**
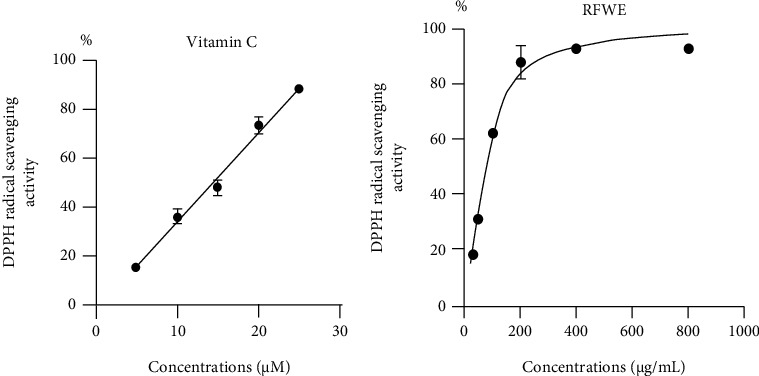
The antioxidant capacity of rumdul fruit water extract (RFWE). (a) DPPH radical scavenging activity of the vitamin C, *R*^2^ = 0.9846, IC_50_ = 14.49 ± 1.08 *μ*M. (b) DPPH radical scavenging activity of RFWE, *R*^2^ = 0.9947, IC_50_ = 85.62 ± 1.05 *μ*g/mL. Data represent means and the standard deviation (SD).

Previous reports demonstrated that antioxidant compounds could ameliorate PD-like symptoms in *dUCH*-knockdown fly model [9] [10]. We thereby examined the antioxidant capacity of rumdul fruit water extract (RFWE) by DPPH radical scavenging activity assay in which vitamin C (vitC) was used as the standard compound. The results showed that the IC_50_ value of RFWE was 85.62 ± 1.05 *μ*g/mL (Figure 1(b)), equivalent to an antioxidant capacity of 14.49 ± 1.08 *μ*M vitamin C (Figure 1(a)). Based on the equivalent antioxidant index of RFWE and vitamin C (presented in Table S1) and a previous report on the effect of vitC treatment [9], we decided to use RFWE at concentrations of 3, 6, 12, and 18 mg/mL for further experiments on *dUCH*-knockdown fly model.

### 3.2. RFWE Concentration-Dependent Food Intake and Relative Antioxidant Intake

**Figure 2:**
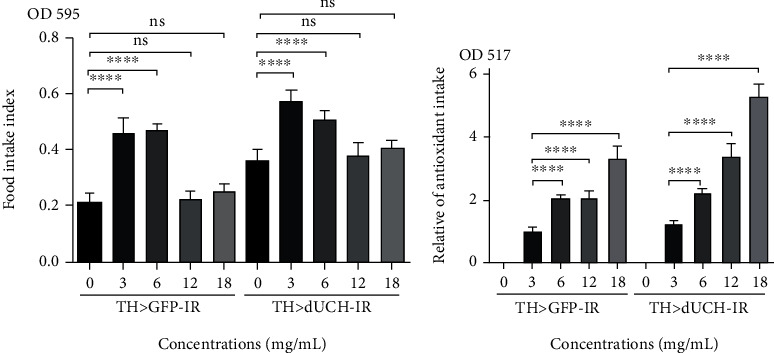
RFWE concentration-dependent food intake and the relative antioxidant intake. (a) Effect of concentrations of RFWE on food intake of third-instar larvae. The control strain TH>*GFP*-IR (+; UAS-*GFP*-IR; TH-GAL4) and the *dUCH*-knockdown strain TH>*dUCH*-IR (+; +; TH-GAL4/UAS-*dUCH*-IR). Population size *N* = 10 and biological replication *n* = 8, one-way ANOVA, Dunnett's multiple comparisons test (ns: not significant, ^∗∗∗∗^*p* < 0.0001. Data are means ± SD). (b) Effect of concentrations of RFWE on antioxidant intake. The control line TH>*GFP*-IR and the *dUCH*-knockdown line TH>*dUCH*-IR. Population size *N* = 10 and biological replication *n* = 8, one-way ANOVA, Turkey's multiple comparisons test (ns: not significant, ^∗∗∗∗^*p* < 0.0001. Data are means ± SD).

Food intake is one of the important criteria to evaluate the effectiveness of the treatment by oral route. The abnormal food intake may lead to inaccurate assessments of the effect as well as the dose of extracts. In this study, the changes of fly medium when it was supplemented with rumdul extract might affect the fly's food intake. Therefore, to address whether the RFWE has effects on fly nutrient absorption, feeding assay was carried out on both *dUCH*-knockdown and control fly. The results showed that rumdul extract at concentrations of 3 and 6 mg/mL increased the amount of consumed food 2.2 times in the control group (^∗∗∗∗^*p* < 0.0001, one-way ANOVA) while that is 1.6 and 1.4 times, respectively, in the *dUCH*-knockdown group (^∗∗∗∗^*p* < 0.0001, one-way ANOVA). However, there were no differences in food intake at the concentrations of 12 and 18 mg/mL (Figure 2(a)) (ns, one-way ANOVA).

According to the results of food intake, we estimated the antioxidant intake of both fly strains. Compared to the 3 mg/mL RFWE supplemented medium, 6 mg/mL RFWE medium increased the amount of antioxidant compound to 2.0 and 1.8 times in control and knockdown flies, respectively (^∗∗∗∗^*p* < 0.0001, one-way ANOVA). It is noted that, although the RFWE at the concentration at 12 and 18 mg/mL did not have an influence on the flies' feeding behavior, they also elevated the antioxidant intake to 2.0 and 3.3 times in control flies and 2.7 and 4.3 times in knockdown flies, respectively (^∗∗∗∗^*p* < 0.0001, one-way ANOVA). Moreover, in comparison to control larvae fed on the same medium condition, the *dUCH*-knockdown larvae at both concentrations of 12 and 18 mg/mL had 1.6 times higher antioxidant intakes (Figure 2(b)).

Taken together, RFWE had a strong effect on food intake of the fly and thereby significantly altered the antioxidant intake in fly strains.

### 3.3. Effect of Rumdul Fruit Water Extract RFWE on Drosophila Melanogaster Development and Life Span

One of the most important features to be considered in drug screening is their side effects. We, therefore, evaluated the effects of RFWE on *Drosophila* development and life span.

**Figure 3:**
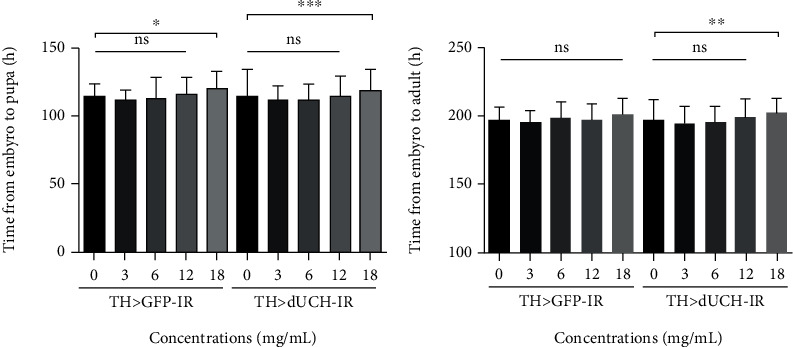
Effects of rumdul fruit extract on *Drosophila melanogaster* development. Control strain (TH>*GFP*-IR) and *dUCH*-knockdown strain (TH>*dUCH*-IR). (a) Period from embryo stage to pupa stage. (b) Period from embryo stage to adult stage. (*n*_A_ = 91, *n*_B_ = 89; one-way ANOVA, Kruskal-Wallis test, Dunn's multiple comparison test, ^∗^*p* < 0.05, ^∗∗^*p* < 0.01, and ^∗∗∗^*p* < 0.001). Data are means ± SD.

Our data revealed that using RFWE at the concentration of 3-12 mg/mL did not affect the development speed of both fly strains in every development stage. However, RFWE at 18 mg/mL prolonged the larva stage of both *dUCH*-knockdown flies and control flies (Figure 3(a)). However, counting on the period of embryo to adult stage, 18 mg/mL concentration of RFWE affected *dUCH* knockdown flies only (Figure 3(b)).

**Figure 4:**
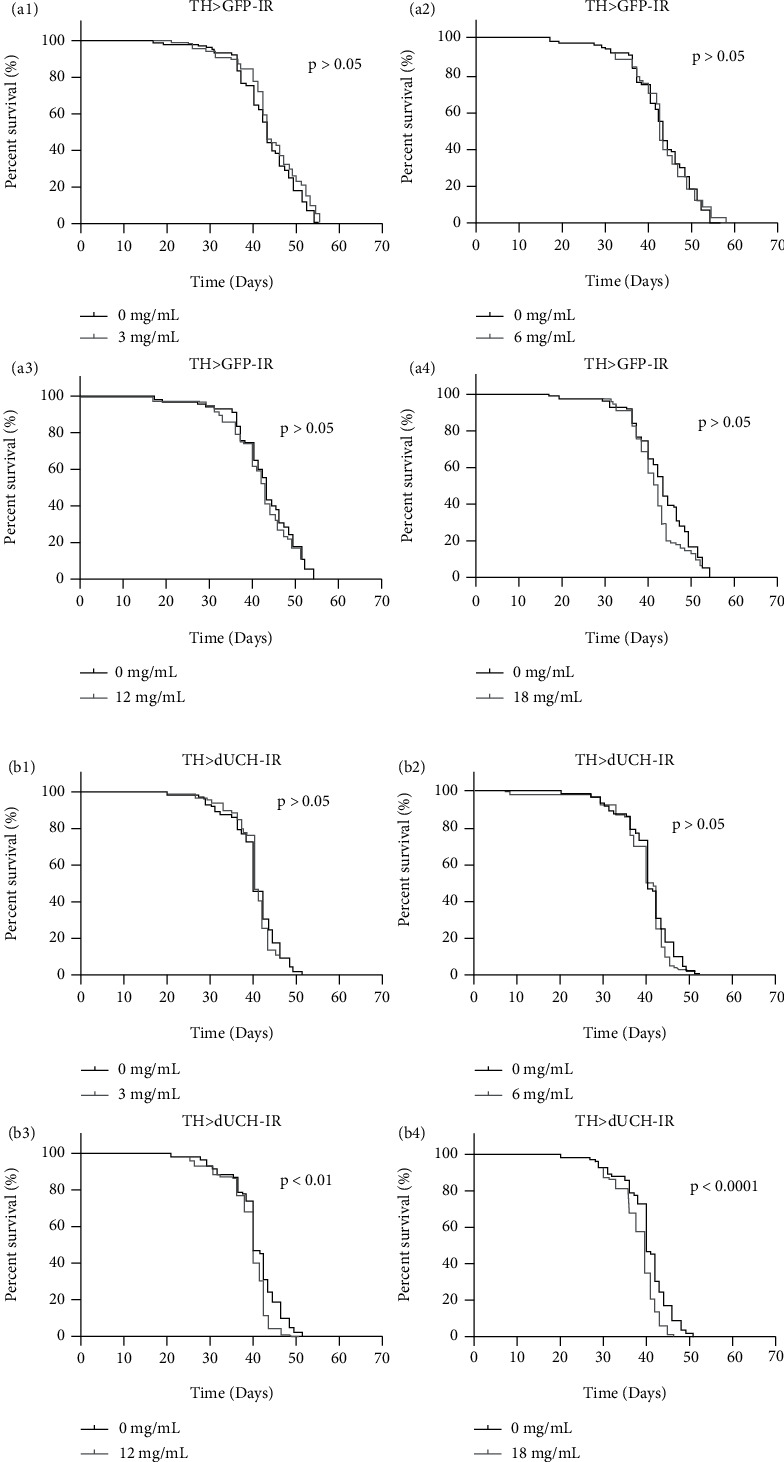
The effect of RFWE on survival of control and *dUCH*-knockdown flies. (a_1_–a_4_) RFWE did not have an impact on survival curves of control flies (TH>*GFP*-IR) ((a_1_–a_4_) display comparisons between concentrations of 3, 6, 12, and 18 mg/mL, separately, and 0 mg/mL). (b_1_–b_4_) RFWE shortened the survival curves of *dUCH*-knockdown flies at concentrations of 12 and 18 mg/mL (TH>*dUCH*-IR) (b_1_–b_4_ display comparisons between concentrations of 3, 6, 12, and 18 mg/mL, separately, and 0 mg/mL). Population size *N* = 100. Log-rank (Mantel-Cox) test. Data are percent survival at each day.

Besides the observation of *Drosophila* development, we further evaluated the life spans of *Drosophila* in RFWE utilization. Consistent to the observation of *Drosophila* development, treatment with RFWE at 18 mg/mL shortened life spans of both fly strains (Figures 4(a_4_) and 4(b_4_) . However, the RFWE at 12 mg/mL only reduced the life span of adult knockdown flies (^∗∗^*p* < 0.01, Log-rank (Mantel-Cox test)). These results suggested that knockdown *dUCH* flies were more vulnerable to the RFWE.

It is interesting to note that there was no discernible difference in vitality when treating flies with high concentration (18 mg/mL) in the initial period. The half-life of both treated and untreated flies was 40 days. However, after that, the proportion of untreated survival knockdown flies dropped substantially, so that on the 46^th^ day, the number of survival flies was 10%, compared to the 43^rd^ day in RFWE-treated flies. These results strongly indicated that using a high amount of RFWE could induce unwanted effects on *Drosophila* physiology, and these impacts were accumulated throughout the time.

### 3.4. Rumdul Fruit Water Extract Ameliorated Locomotor Dysfunction Caused by Knockdown of dUCH

DA-specific knockdown *dUCH* fly model mimicked PD symptoms in which motor dysfunction was displayed [8]. Moreover, treatment with vitamin C at 0.5 mM was able to rescue the PD-like symptoms at third-instar larvae and its treatment potential was lost at 2.5 mM and 5 mM [9]. Here, we conducted experiments to evaluate the effects of REWE at the concentration of 3, 6, 12, and 18 mg/mL on locomotor ability of third-instar larvae.

**Figure 5:**
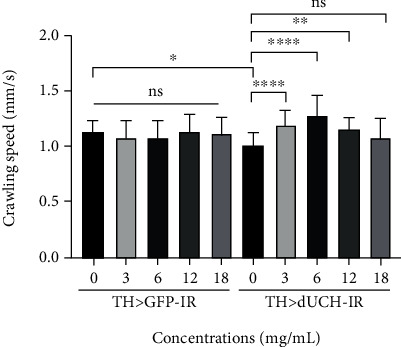
Effect of rumdul fruit water extract on motor PD-like symptoms of the *dUCH-*knockdown model. RFWE ameliorated the motor impairment of third-instar larvae at the concentrations of 3, 6, and 12 mg/mL. Control strain TH > *GFP* − IR (*+*; *UAS-GFP-IR*; *TH-GAL4*) and *dUCH*-knockdown strain TH > *dUCH* − IR (*+*; *+*; *TH-GAL4/UAS-dUCH-IR*). Population size *n* = 33, one-way ANOVA, Turkey's multiple comparisons test, ns: not significant, ^∗^*p* < 0.05, ^∗∗^*p* < 0.01, and ^∗∗∗^*p* < 0.001. Data are means ± SD.

Our results showed that, in the larval stage, REWE had no significant effect on the crawling ability of control flies. Otherwise, it had a strong effect on *dUCH* knockdown flies. We demonstrated that RFWE at the concentrations of 3, 6, and 12 mg/mL improved the mobility of *dUCH*-knockdown larvae (crawling speeds 1.20, 1.29, and 1.16 mm/s, respectively), compared with untreated knockdown larvae (crawling speeds 1.01 mm/s) (one-way ANOVA) (Figure 5). However, the effects decreased at high concentration; in detail, there is no improvement when *Drosophila* was treated with 18 mg/mL RFWE (Figure 5).

### 3.5. Rumdul Fruit Water Extract Rescued the Degeneration of Dopaminergic Neurons in *dUCH*-Knockdown Fly

Previous studies showed that DA neuron-specific knockdown of *dUCH* on the *Drosophila* model caused random degeneration of DA neurons in different clusters and treating with antioxidant could rescue this effect [8]. Therefore, we treated the *dUCH* knockdown model of PD with RFWE and examined number of DA neurons in the treated fly by immunostaining with tyrosine hydroxylase antibody—an enzyme specific for the dopamine synthesis process.

**Figure 6:**
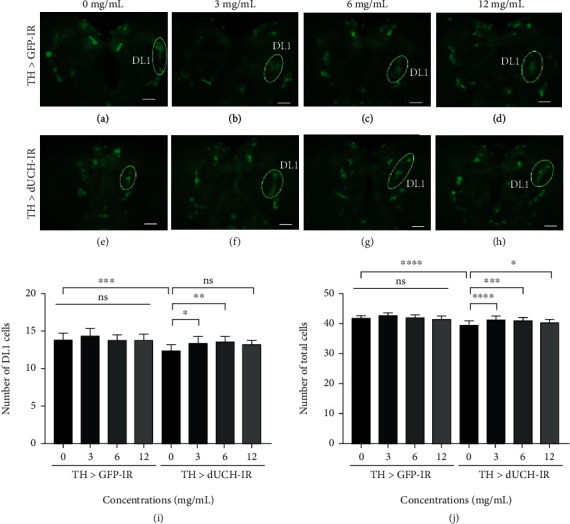
Rumdul fruit water extract rescued degeneration of dopaminergic neurons caused by knockdown of *dUCH* in larvae. (a–h) Immunostaining image of brain lobes with anti-tyrosine hydroxylase antibody, a marker for dopaminergic neurons. The control TH>*GFP*-IR (+; UAS-*GFP*-IR; TH-GAL4) and *dUCH*-knockdown larvae TH>*dUCH*-IR (+; +; TH-GAL4/UAS-*dUCH*-IR) were treated with 0, 3, 6, and 12 mg/mL of RFWE. Scale bars indicate 50 *μ*m. (i, j) The quantified data described the number of dopaminergic neurons in clusters ((i) DL1, (j) sum of three clusters including DL1, DL2, and DM) (population size *N* = 10; one way ANOVA, post hoc test: Dunnett, ns: not significantly, ^∗^*p* < 0.05, ^∗∗^*p* < 0.01, and ^∗∗∗^*p* < 0.001). Data are means ± SD.

We found that *dUCH*-knockdown untreated larvae had the severe loss of DA neurons in the DL1 cluster (Figures 6(e) and 6(i)) and the total number of DA neurons (Figure 6(j)). This result was consistent with the previous study [12, 14]. RFWE did not have an impact on the number of DA neurons in the control group at all experimental concentrations (Figures 6(b)–6(d), 6(i), and 6(j)).

As expected, when the *dUCH*-knockdown larvae were treated with RFWE at concentrations of 3 and 6 mg/mL, the loss of DA neurons was reduced, in DL1 cluster and all total DA neurons also (Figures 6(f)–6(i)). These results were consistent with the improvement in the motor function of *dUCH-*knocked down flies. However, at a concentration of 12 mg/mL, RFWE could not significantly prevent the loss of DA neurons in the DL1 cluster (Figures 6(h) and 6(i)).

**Figure 7:**
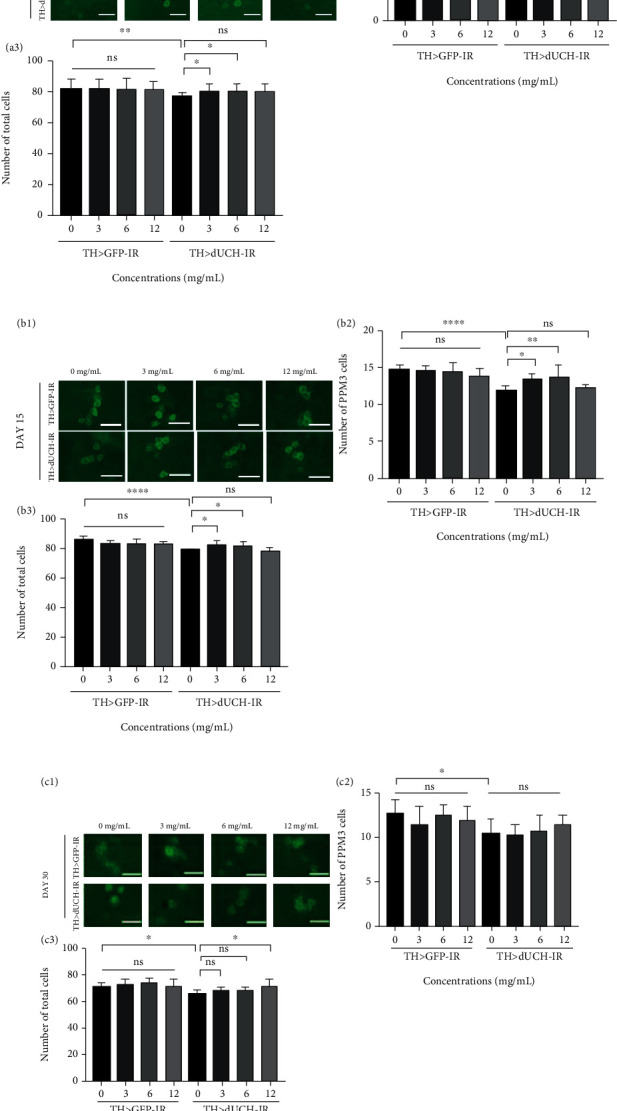
Rumdul fruit water extract ameliorates the degeneration of dopaminergic neurons caused by knockdown of *dUCH* in adult flies. (a_1_–c_1_) Immunostaining images of dopaminergic neurons of the control (TH>*GFP*-IR) and *dUCH*-knockdown flies (TH>*dUCH*-IR) treated with 0, 3, 6, and 12 mg/mL of RFWE at day 1 (a_1_), day 15 (b_1_), and day 30 (c_1_). Scale bars indicate 20 *μ*m. (a_2_–c_2_) The numbers of dopaminergic neurons in the PPM3 cluster at 1-day-old (a_2_), 15-day-old (b_2_), and 30-day-old (c_2_) (*n* = 7, *n* = 10, and *n* = 19, respectively). (a_3_–c_3_) The total numbers of dopaminergic neurons at 1-day-old (a_3_), 15-day-old (b_3_), and 30-day-old (c_3_) (*n* = 7, *n* = 10, and *n* = 19, respectively). One-way ANOVA, post hoc test: Dunnett, ns: not significant, ^∗^*p* < 0.05, ^∗∗^*p* < 0.01, and ^∗∗∗∗^*p* < 0.0001. Data are means ± SD.

At the adult stage, the *dUCH* knockdown triggered the death/degeneration of DA neurons, which could be observed in the reduction of both DA numbers in PPM3 clusters (from 1 to 15 day-age) (Figures 7(a_1_)–7(a_2_),and 7(b_1_)–7(b_2_)) and total DA neurons (from day 1 to day 30) (Figures 7(a_3_)–7(c_3_)). Moreover, similar with the larval stage, using RFWE in the range from 3 to 12 mg/mL did not have any impact on DA of adult control flies (Figure 7) (ns, *p* < 0.05, one-way ANOVA).

For the *dUCH* knockdown flies, RFWE at the concentration of 3 mg/mL was able to protect DA neurons of the PPM3 cluster and prevent the loss/degeneration of DA neurons from day 1 to day 15 (Figures 7(a_1_)–7(a_2_),and 7(b_1_)–7(b_2_)). At 6 mg/mL, RFWE did not show significant effectiveness in rescuing DA neurons in the PPM3 cluster, except on day 15 (Figures 7(a_1_), 7(a_2_), 7(b_1_), and 7(b_2_)), but overall, it helped to improve the total number of DA on 1-day-old and 15-day-old knockdown flies (Figures 7(a_3_)–7(b_3_)). However, the amelioration of DA loss was not observed at 30 days old (Figure 7(c_3_). Interestingly, RFWE at 12 mg/mL showed the amelioration of DA lost when it was used for up to 30 days (Figures 7(a_3_)–7(c_3_)).

Taken together, our results showed that RFWE could ameliorate the death/degeneration of DA neurons in *dUCH*-knocked down flies at suitable concentration and used time.

## 4. Discussion

Rumdul belongs to the *Annonaceae* family, which was reported as a high antioxidant capacity plant due to the high level of phenolic compounds and ascorbic acid [16] [17]. A previous study proposed that the antioxidant capacity of rumdul was higher than that of other rich oxidative fruit such as apple [13]. So far, none of the studies has reported on the pharmaceutical activities of rumdul. In this study, our results for the first time demonstrated that water extract of rumdul fruit (RFWE) has high potential for Parkinson's disease (PD) treatment due to the amelioration of PD-like symptoms on the *Drosophila* PD model.

Treatment by RFWE at 3 mg/mL-12 mg/mL could reduce the defect on mobility at the larva stage, and using it at 6 mg/mL could give the best outcome (Figure 5). This result is highly consistent with the amelioration of DA loss in RFWE-treated flies at the same concentrations (Figure 6).

However, RFWE utilization during the time from the larvae to the late adult stage could decrease their treatment effectiveness. In detail, up to 15-day-old, RFWE could prevent the death/degeneration at 3 mg/mL and 6 mg/mL, but they did not show any impact on the 30-day-old knockdown flies (Figure 7). This result implied that using RFWE for a long time induced some side effects or triggered toxic accumulation in *Drosophila melanogaster*, thereby limiting their pharmaceutical activities. The result might also imply that at elder stage, deterioration of locomotion and DA loss of fly also accumulated by age; thereby, the effect of RFWE might be influenced.

Interestingly, RFWE at the concentration of 12 mg/mL did not perform their treatment ability at the adult stage unless we continued to use it until 30-day-old (Figure 7). Although this phenomenon needs further evidence to be clarified, we would not rule out the possibility that for progressive diseases like PD, at the severe stage, it is necessary to use high concentrations of RFWE to see an improvement.

RFWE effects could partly come from their supplying antioxidant ability. In the result of the DPPH free radical assay, we reported that IC_50_ of RFWE was 85.62 ± 1.05 *μ*g/mL, which is equivalent to 14.49 ± 1.08 *μ*M vitamin C (Figure 1). Previous studies proposed that rumdul fruits have a great antioxidant capacity due to the high level of ascorbic acid and phenolic compounds [13]. Ascorbic acid is a powerful antioxidant having an ability to donate a hydrogen atom and form a relatively stable acerbity free radical. It is a water-soluble molecule, so it neutralizes free radicals and prevents free radical damage both inside and outside the cells. Ascorbic acid can neutralize almost ROS generated from cellular activities such as superoxide (O2-), hydroxyl (^·^OH), or hydrogen peroxide (H_2_O_2_) [18]. The results matched well with previous studies which showed the potential of plant extracts that contained phenolic compound such as *Ginseng*, *Mucuna pruriens*, and *Tinospora cordifolia* in PD treatment [19] [20] [21] [22].

Our results also showed that 3 mg/mL and 6 mg/mL RFWE supplemented food provided higher nutrient and antioxidant ability compared to the standard medium, thanks to the increasing food intake and RFWE antioxidant compounds (Figure 2). Moreover, the absorbed antioxidant is accessed more and more when being treated at high concentrations. Despite the unchanged food intake at the concentrations at 12 mg/mL and 18 mg/mL, the antioxidant accumulation still significantly increased. Because the PD symptoms on *dUCH-*knockdown might be induced by oxidative stress [5], RFWE could partly rescue these symptoms by their antioxidant activity. This phenomenon was similar with other reported antioxidants such as vitamin C, curcumin, or *Portulaca oleracea* [9] [10] [11]. Besides that, for the high RFWE concentration, the side effect on flies could be originated partly from the imbalance between the nutrient and antioxidant amount and antioxidant overdoses, which has been mentioned in previous studies [17] [23] [24].

The hypothesis about the side effects of RFWE, when being used for a long time and high concentration, was supported by our results of RFWE's influence on the flies' physiology. In detail, our results showed that using 18 mg/mL RFWE slowed down the development speed of *Drosophila* in both knockdown and control flies (Figure 3). Moreover, their side effects did not completely occur at an early stage but over time, by the gradual toxin accumulation. It could be one of the reasons why RFWE triggered a negative impact on *Drosophila* vitality at the concentration of 12 mg/mL and 18 mg/mL, and it is worthy to note that the *dUCH*-knockdown flies were more vulnerable to RFWE than the control flies (Figure 4). These mentioned results were also consistent with other reports, which proposed the producing neurotoxicity and growth inhibition of fruits belonging to the *Annonaceae* family when absorbed by the gastrointestinal tract [25] [26]. Besides, as recent reports mentioned about promising drug target, our study contributed one more piece for a whole picture of advancement in modelling and associated therapeutic of PD [27] [28].

## 5. Conclusion

In conclusion, our results strongly demonstrated that rumdul fruit, with its high antioxidant activity, is a potential candidate for developing PD treatment products. They could rescue the fundamental symptoms on *dUCH*-knockdown flies and did not show serious problems on control flies. However, the dose and time of rumdul utilization need to be further studied to figure out the best usage condition and minimize their side effects.

## Data Availability

We state that the underlying data supporting the results of our study can be found, including, where applicable, hyperlinks to publicly archived datasets analyzed or generated during the study.

## Supplementary Materials

Supplementary Materials Table S1: vitamin C equivalent antioxidant capacities of the RFWE.
