# Supplementary material for: Rumdul (Sphaerocoryne affinis) Antioxidant Activity and Its Potential for Parkinson's Disease Treatment
Source: Oxid Med Cell Longev. 2022 Mar 18;2022:8918966. doi: 10.1155/2022/8918966 (PMC8956402; doi:10.1155/2022/8918966)
Supplement: Supplementary Materials — Table S1: vitamin C equivalent antioxidant capacities of the RFWE. [file 8918966.f1.docx]

**Supplementary Materials**

**Table S1.** Vitamin C equivalent antioxidant capacities of the RFWE

| Concentrations of RFWE  (mg/ml) | Vitamin C equivalent antioxidant capacities (VCEAC) (mM ± SE) |
| --- | --- |
| 3 | 0.51 ± 0.04 |
| 6 | 1.02 ± 0.09 |
| 12 | 2.03 ± 0.18 |
| 18 | 3.05 ± 0.26 |
